# Prediction of anti-inflammatory proteins/peptides: an insilico approach

**DOI:** 10.1186/s12967-016-1103-6

**Published:** 2017-01-06

**Authors:** Sudheer Gupta, Ashok K. Sharma, Vibhuti Shastri, Midhun K. Madhu, Vineet K. Sharma

**Affiliations:** Metagenomics and Systems Biology Group, Department of Biological Sciences, Indian Institute of Science Education and Research Bhopal, Bhopal, India

## Abstract

**Background:**

The current therapy for inflammatory and autoimmune disorders involves the use of nonspecific anti-inflammatory drugs and other immunosuppressant, which are often accompanied with potential side effects. As an alternative therapy, anti-inflammatory peptides are recently being exploited as anti-inflammatory agents for treatment of various inflammatory diseases such as Alzheimer’s disease and rheumatoid arthritis. Thus, understanding the correlation between amino acid sequence and its potential anti-inflammatory property is of great importance for the discovery of novel and efficient anti-inflammatory peptide-based therapeutics.

**Methods:**

In this study, we have developed a prediction tool for the classification of peptides as anti-inflammatory epitopes or non anti-inflammatory epitopes. The training was performed using experimentally validated epitopes obtained from Immune epitope database and analysis resource database. Different sequence-based features and their hybrids with motif information were employed for development of support vector machine-based machine learning models. Similarly, machine learning models were also constructed using random forest.

**Results:**

The composition and terminal residue conservation analysis of peptides revealed the dominance of leucine, serine, tyrosine and arginine residues in anti-inflammatory epitopes as compared to non anti-inflammatory epitopes. Similarly, the anti-inflammatory epitopes specific motifs were found to be rich in hydrophobic and polar residues. The hybrid of tripeptide composition-based support vector machine model and motif yielded the best performance on 10-fold cross validation with an accuracy of 78.1% and MCC of 0.58. The same displayed an accuracy of 72% and MCC of 0.45 on validation dataset, rejecting any possibility of over-fitting. The tripeptide composition-based random forest model displayed an accuracy of 0.8 and MCC of 0.59 on 10-fold cross validation, however, the accuracy (0.68) and MCC (0.31) was lower as compared to support vector machine model on validation dataset. Thus, the support vector machine model is implemented as the default model and an additional option of using the random forest model is provided.

**Conclusion:**

The prediction models along with tools for epitope mapping and similarity search have been provided as a web server which is freely accessible at http://metagenomics.iiserb.ac.in/antiinflam/.

**Electronic supplementary material:**

The online version of this article (doi:10.1186/s12967-016-1103-6) contains supplementary material, which is available to authorized users.

## Background

For maintenance of immune homeostasis and restricting the occurrence of aggravated inflammation and autoimmunity, the induction of immune tolerance is crucial [1]. The effect of multiple mechanisms working in concert is necessary for the upkeep of the state of tolerance. Several endogenous peptides identified during inflammatory responses have emerged as anti-inflammatory agents that can be employed in new therapies for inflammatory and autoimmune conditions [2]. The immunotherapeutic ability of these anti-inflammatory peptides in the inhibition of antigen-specific T(H)1-driven responses, and in the generation of regulatory T cells has numerous clinical applications [3].

Several synthetic and natural peptides are known to inhibit the signal transduction pathways for expression of inflammatory cytokines. For example, intranasal treatment of amyloid-beta (Abeta) peptide in mice with neuropathological symptoms of Alzheimer’s disease showed a decreased A-beta plaque burden along with anti-inflammatory cytokines IL-4, IL-10 and tumor growth factor-beta [4]. Another neuropeptide: vasoactive intestinal peptide (VIP) is also known to reduce the inflammatory and autoimmune components of rheumatoid arthritis in the experimental model [5]. Similarly, there are pieces of evidence showing production of pro-inflammatory cytokines and microglial activation during age-related cognitive deficits in the hippocampal region. A mimetic peptide FG Loop(FGL) derived from the neural cell adhesion molecule (NCAM) is shown to work as a novel anti-inflammatory agent in such age-related disorders [6]. Furthermore, nonapeptides, Anti inflammin 1 and 2, can potentially reduce leukocyte trafficking in host defense and inflammation by inhibiting PMN adhesion to HCAEC by attenuating up-regulation of CD11/CD18 expression on leukocytes [7]. Moreover, certain synthetic peptides are known to be involved in inhibition of early signal transduction pathways of inflammatory cytokines. One notable example is the role of intravesical RDP58 in interstitial cystitis (IC) inflammation-induced neurotrophic changes in the bladder. Mice models treated with RDP58 shows near complete abolition of LPS induced production of TNF-α in the bladder [8].

Taken together, the availability of experimental data allows for the computational correlation between amino acid sequences and their anti-inflammatory properties. To the best of the authors’ knowledge, there has been no study that investigates the sequence based signature for the anti-inflammatory behavior of target peptide sequences. Understanding the association between specific amino acid sequences and the anti-inflammatory properties conferred by them is likely to facilitate their application for therapeutic purposes. One such example is of altered peptide ligands (APLs) and their role in therapeutic modulation of T cell function [9]. The aim of this study was to examine the amino acid sequences of experimentally validated anti-inflammatory epitopes (AIEs) and non anti-inflammatory epitopes (NAIEs) and develop a machine learning based classification method that incorporates the sequence based features to predict the anti-inflammatory nature of various peptides and proteins.

## Methods

### Preparation of dataset

**Fig. 1:**
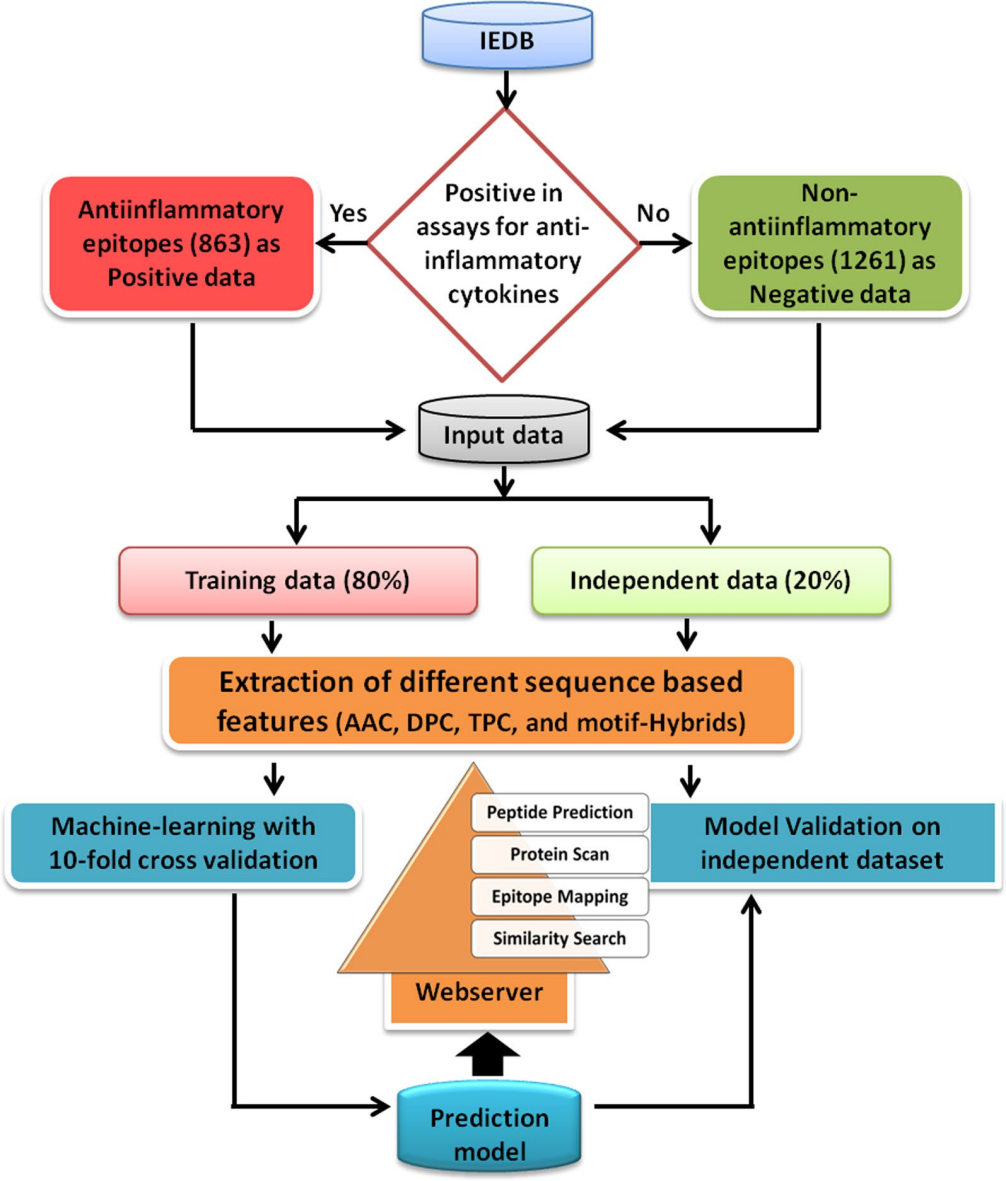
Flowchart showing steps involved in the development of prediction model and web server

An unambiguous and well-curated dataset is required for machine-learning based model building. Thus, the positive and negative assaying epitopes were retrieved from the immune epitope database [10]. A peptide was considered as anti-inflammatory if it was shown to induce any one of the anti-inflammatory cytokines [IL-10, IL-13, IL-22, IL-1RA, TGFβ, IFN-α/β] [11]. 863 epitopes were reported to show a positive assay, whereas, 1261 peptides tested negative for anti-inflammatory cytokines and were classified as NAIEs. The length of these epitopes ranged between 4 and 30 amino acids residues. Out of the total input dataset, 80% of both AIEs and NAIEs were assigned as training dataset and the remaining 20% constituted the validation dataset. Resultantly, a total of 690 AIEs (positive data) and 1009 NAIEs (negative data) constituted the training dataset, and the validation dataset contained 173 AIEs (positive data) and 253 NAIEs (negative data) (Fig. 1). Two datasets were constructed on which the various analysis were carried out in this manuscript, namely, (1) realistic dataset with all available positive and negative epitopes, and (2) balanced dataset including all positive epitopes (690 positives) and randomly chosen equal number of negative epitopes (690 negatives).

### Input features for machine learning

#### Composition-based features

##### Amino acid composition

Amino acid composition (AAC) is a widely used feature in composition-based peptide classification methods [12, 13]. It is the percentage of each amino acid in a given length of the peptide sequence. Each peptide/protein can be represented as a vector of 20 features (composition of 20 naturally occurring amino acids).

$${\text{AAC}}({\text{i}}) = \frac{\text{Total number of amino acid (i)}}{\text{Total number of all possible amino acids }} \times 100$$

where, AAC is the amino acid composition of an amino acid (i) which is one of the 20 amino acids.

##### Dipeptide composition

Several immune epitope prediction algorithms and numerous composition-based algorithms use dipeptide composition (DPC) for classification [14, 15]. DPC is the percentage composition of each of the 400 possible dipeptides formed by 20 amino acids. Unlike AAC, DPC carries the additional information of local arrangement in a peptide/protein since it is the pair of adjacently placed amino acids. DPC was calculated using the following equation.

$${\text{DPC(i)}} = \frac{\text{Total number of dipeptides (i)}}{\text{Total number of all possible dipeptides }} \times 1 0 0$$

where Dp(n) is a dipeptide n out of 400 dipeptides.

##### Tripeptide composition

Tripeptide composition (TPC) is the percentage composition of each of the 8000 possible dipeptides formed by 20 amino acids and was calculated using the following formula. It can also be used for composition-based classification [16].

$${\text{TPC(i}}) = \frac{\text{Total number of tripeptides (i)}}{\text{Total number of all possible tripeptides}} \times 1 0 0$$

where TPC(n) is a tripeptide n out of 8000 tripeptides.

### Motif-based features

Identification of functional motifs in amino acid sequences is one of the widely used techniques in the functional annotation of peptides/proteins. Several previous immunoinformatics studies have examined the immunologically relevant motifs in peptides for various purposes [17–19]. To identify the motifs specific to AIEs by comparing it with NAIEs, MERCI software was used [20]. The identification of motifs in positive data by comparing with negative data yields motifs exclusive for the positive data. Motifs specific for NAIEs were also identified by MERCI software in a similar manner. The maximum length of the motif was set to 9 amino acids because the core size of both MHC I and II equals nine amino acids [21, 22]. The gap length was set to the default length of 1.

### Hybrid feature

Hybrid models of AAC, DPC, and TPC with MERCI-based motifs were also constructed in this study [23, 24]. The presence of anti-inflammatory and non anti-inflammatory specific motifs in every peptide in the training dataset was examined. If the epitope contained the anti-inflammatory motifs, a weightage of +1 was added to its AAC, DPC or TPC based SVM score. Similarly, if the epitope is positive for non anti-inflammatory motifs, the weight was added as −1 for the same.

### Machine-learning-based prediction models

Caret package in R was used to evaluate the performance of various machine learning methods such as support vector machine (SVM), Random Forest (RF), *k*-nearest neighbors (kNN), linear discriminant analysis (LDA) and classification and regression tree (CART). Based on the results, SVM and RF were used as the machine learning algorithms for building predictive models through SVM^light^ package available at http://svmlight.joachims.org/ and randomForest package available at https://cran.r-project.org/web/packages/randomForest/index.html. Machine learning algorithms have also been used in previous studies for developing tools for immune epitope prediction with good performances [15, 25–28].

### Performance evaluation of models

The comparison of different machine learning methods to select the best method is essential in predictive modelling. A 10-fold cross-validation technique was employed for different learning methods and evaluated on a separate unseen validation dataset. To carry out the 10-fold cross-validation, the data was divided into ten parts of which nine parts were used for training and the tenth segment was used for testing. The procedure is repeated ten times to test the learning methods on each part. The result of all ten predictions was taken together for evaluating the performance of various models built by different methods. For measuring the performance of each model, threshold dependent and threshold independent parameters were used. For all models, area under curve (AUC), a threshold independent parameter was calculated using PERF [29]. Threshold-dependent parameters such as sensitivity (Sen), specificity (Spec), accuracy (Acc) and Matthews’s correlation coefficient (MCC) were also calculated for all models. The following equations were used for calculating the threshold dependent parameters.

In addition, a fivefold cross validation study was also carried out and performance was compared with the 10-fold cross validation study.

$${\text{Accuracy}} = \frac{{{\text{TP}} + {\text{TN}}}}{{{\text{TP}} + {\text{FN}} + {\text{FP}} + {\text{TN}}}}$$

$${\text{Sensitivity}}\;{ = }\;\frac{\text{TP}}{{{\text{TP}} + {\text{FN}}}}$$

$${\text{Specificity}}\; = \;\frac{\text{TN}}{{{\text{TP}} + {\text{FN}}}}$$

$${\text{MCC}}\; = \;\frac{{({\text{TP}} \times {\text{TN}}) {\, -\, }({\text{FP}} \times {\text{FN}})}}{{\sqrt {({\text{TP}} + {\text{FP}})({\text{TP}} + {\text{FN}})({\text{TN}} + {\text{FP}})({\text{TN}} + {\text{FN}})} }} .$$

(TN = true negative, FP = false positive, TP = true positive, FN = false negative; Sens = sensitivity; Spec = specificity; Acc = accuracy)

## Results

### Compositional analysis

**Fig. 2:**
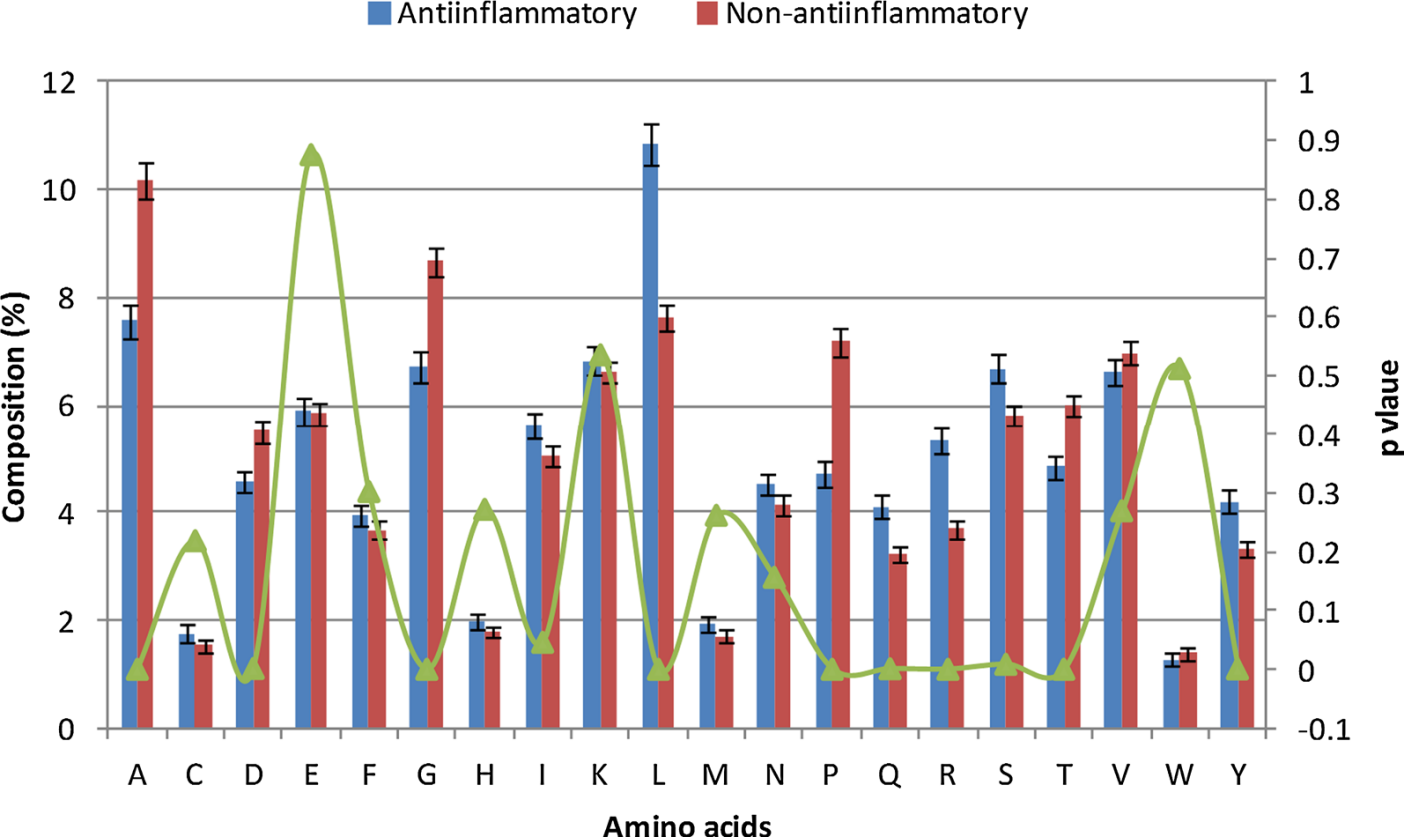
Compositional analysis of AIEs and NAIEs

**Fig. 3:**
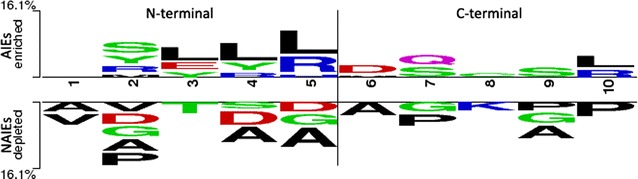
Two sample logo showing positional conservation of five residues at both the terminals (N’- and C’-) in AIEs against NAIEs

To carry out the compositional analysis of AIEs and NAIEs, the frequencies of all amino acids (AAC), dipeptides (DPC), and tripeptides (TPC) were calculated in the peptides included in the two sets. The AAC analysis of AIEs revealed higher abundance of Leu, Ser, Tyr, Gln and Arg (Welch’s t test, p value ≤0.05) (Fig. 2). Similarly for NAIEs, Ala, Gly, Asp, Thr and Pro were observed in higher abundance (Welch’s t test, p value ≤0.05).The positional conservation of amino acids in AIEs and NAIEs was examined using a two sample logo (TSL) analysis (Fig. 3). Leu, Tyr, Ser, Arg and Glu were found as highly conserved at N-terminal of AIEs, whereas, Leu, Gln, Ser and Arg were found at C-terminal. In contrast, Ala, Gly, Val, Pro and Asp were highly conserved at N-terminal, whereas, Pro, Gly and Ala were found conserved at C-terminal of NAIEs.

**Fig. 4:**
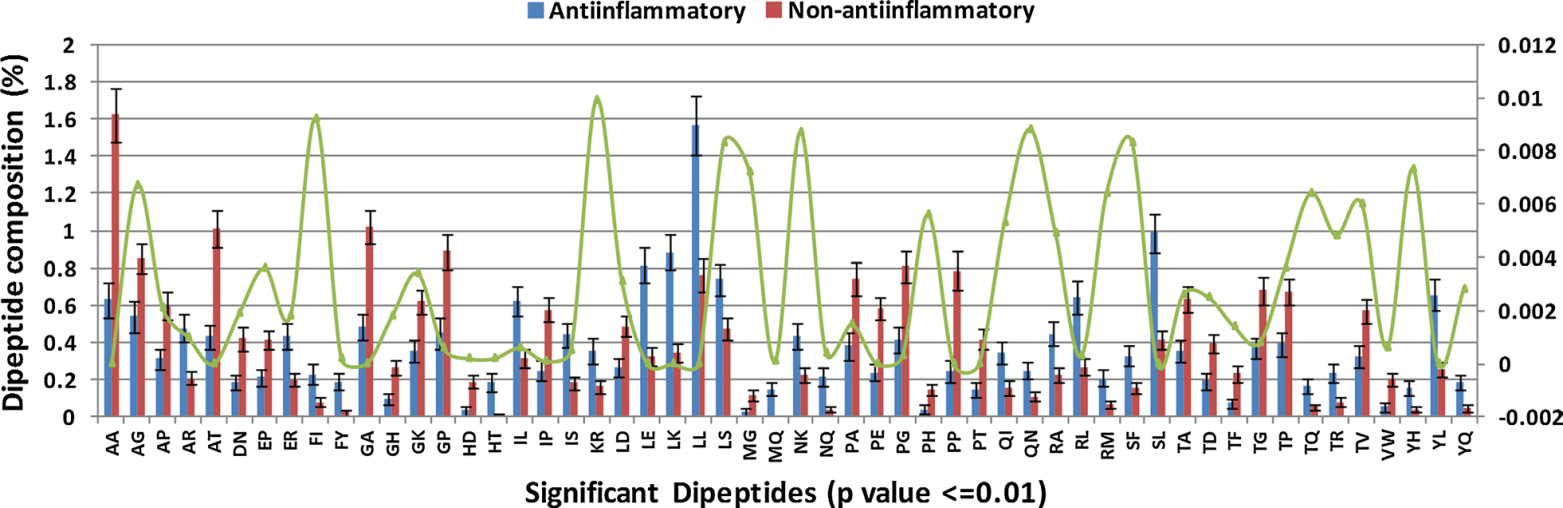
Dipeptide composition distribution between AIEs and NAIEs (only significant dipeptides are shown with p value <0.01 in Welch’s t test)

In DPC, from the 400 possible dipeptides, 54 significant dipeptides (p value ≤0.01, Welch’s t-test) were found significantly different in AIEs as compared to NAIEs (Fig. 4). AIEs were found to be rich in Leu–Leu (LL), Ser–Leu (SL), Leu–Glu (LE), Leu–Lys (LK), Arg–Leu (RL) and Tyr–Leu (YL) dipeptides, whereas, NAIEs were rich in Ala–Ala (AA), Ala–Thr (AT), Gly–Ala (GA), Gly–Pro (GP), Pro–Gly (PG), Pro–Pro (PP), Leu–Pro (LP) and Thr–Gly (TG) dipeptides.

Similarly, the TPC analysis also revealed several tripeptides which were highly abundant in AIEs as compared to NAIEs. A total of 122 tripeptides were found significantly abundant (p value ≤0.01, Welch’s t-test) in AIEs (Additional file 1: Table S1). In particular, Ser–Leu–Ser (SLS), Leu–Lys–Leu (LKL), Leu–Arg–Leu (LRL) etc. are highly abundant in AIEs and their constituent dipeptides and amino acids are also abundant in AAC and DPC analysis.

### HLA allele distribution

**Fig. 5:**
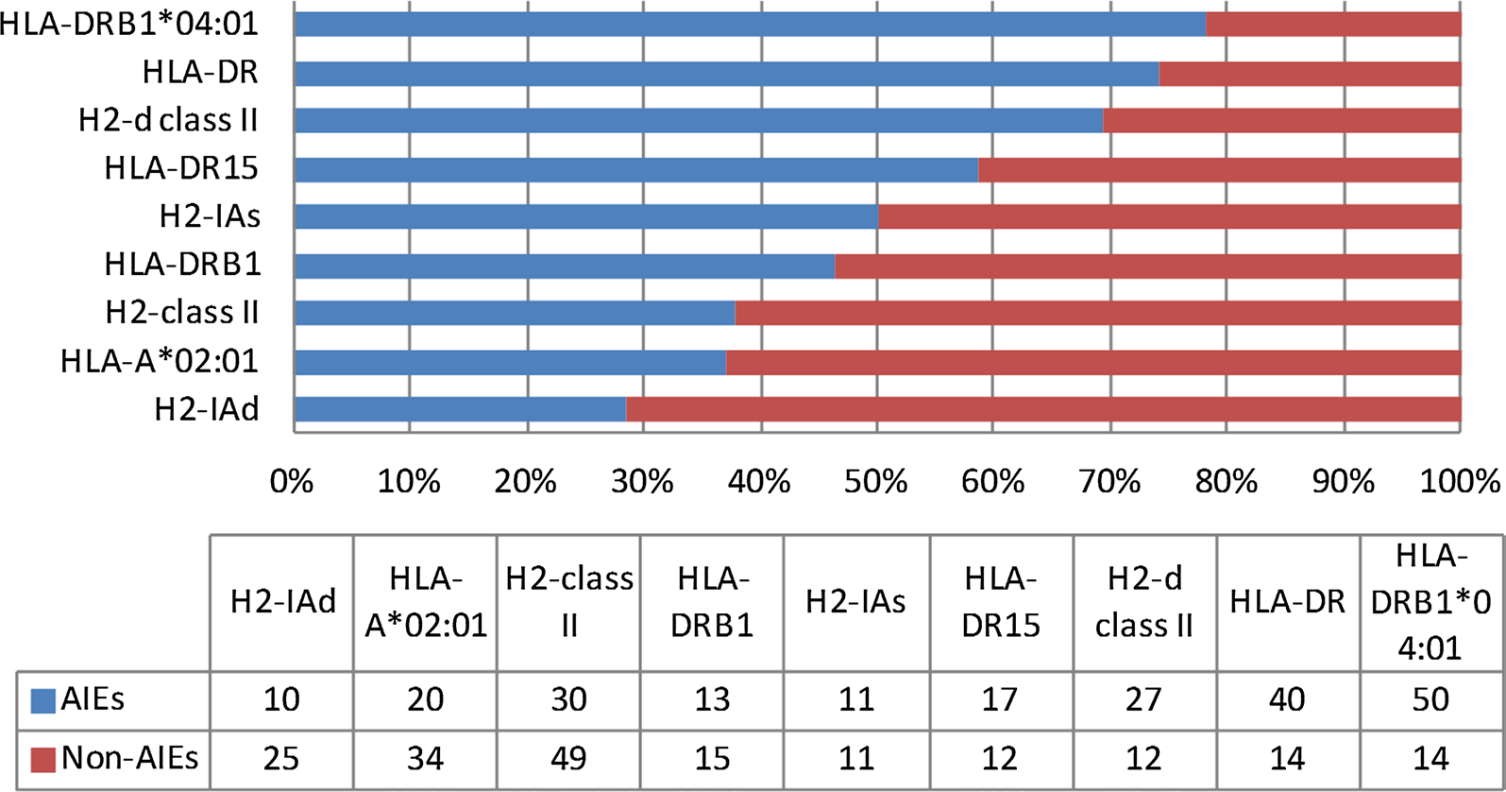
Distribution of HLA alleles among assays reporting AIEs and NAIEs

An analysis of the distribution of epitopes from IEDB database against different HLA alleles was also carried out (Fig. 5). Among the most contrasting results, most (50) of the peptides showed anti-inflammatory response against HLA-DRB1*04:01 in assays, whereas, only 14 peptides showed non anti-inflammatory response against the same HLA allele. Similarly, the assays against H2-IAd allele included 25 unique NAIEs compared to 10 unique AIEs tested against the same HLA allele.

### Motif analysis

Inflammatory responses arise due to the induction of T cells upon activation by antigen-presenting cells. In anti-inflammatory response, MHC molecules on antigen presenting cells present the bounded peptides and initiate the response. Peptide-MHC interaction occurs through specific amino acids in the form of motifs. Thus, the presence of these specific motifs can be utilized as a marker for better classification of peptides. MERCI software was used to predict specific motifs by comparing positive and negative datasets as explained in the “Methods” section. 13 different specific motifs for AIEs were observed as shown in Table 1. The “hydrophobic hydrophobic tiny F charged” motif was found in 22 unique AIEs and had the highest observed motif coverage among all motifs.

**Table 1:** Annotation and coverage of MERCI motifs extracted from anti-inflammatory epitopes

S. no.	Motif	Coverage
1.	Hydrophobic hydrophobic tiny F charged	22
2.	Hydrophobic L hydrophobic hydrophobic small polar hydrophobic polar polar	20
3.	Hydrophobic I hydrophobic hydrophobic hydrophobic hydrophobic hydrophobic polar polar	18
4.	Polar L aliphatic hydrophobic positive	17
5.	Hydrophobic aliphatic hydrophobic aliphatic small polar hydrophobic polar polar	17
6.	Hydrophobic hydrophobic small hydrophobic polar hydrophobic polar Q	17
7.	L hydrophobic aliphatic small polar hydrophobic polar polar	16
8.	Hydrophobic polar hydrophobic L polar hydrophobic hydrophobic polar tiny	16
9.	Hydrophobic L polar polar small small hydrophobic hydrophobic	16
10.	Hydrophobic aliphatic polar small hydrophobic charged polar polar hydrophobic	16
11.	Hydrophobic hydrophobic hydrophobic tiny F charged	16
12.	Hydrophobic hydrophobic tiny F charged hydrophobic	16
13.	small aliphatic E N	16

For example “hydrophobic hydrophobic tiny F charged” motif is found in 22 unique anti-inflammatory epitopes

### Machine learning-based classification

It is apparent from the earlier analysis that AIEs and NAIEs differ in several composition-based features. Therefore, these differences can be exploited for the classification of epitopes. Machine learning-based classification was implemented to exploit the sequence-specific features for the classification and prediction of AIEs and NAIEs.

### Comparison of machine learning methods

Different machine learning algorithms such as SVM, RF, kNN, LDA and CART were implemented using caret package in R for comparing their performances on the same data. Performances of SVM and RF were found better as compared to other machine learning techniques, and hence, these two machine learning methods were optimized further using amino acid, dipeptide and tripeptide composition as input features (Additional file 1: Text S1, Additional file 2: Figure S1, Additional file 3: Figure S2, Additional file 4: Figure S3, Additional file 5: Figure S4, Additional file 6: Figure S5, Additional file 7: Figure S6, Additional file 8: Figure S7, Additional file 9: Figure S8, Additional file 10: Figure S9, Additional file 11: Figure S10 and Additional file 12: Figure S11).

### Performance of SVM models

#### Amino acid composition-based models

**Fig. 6:**
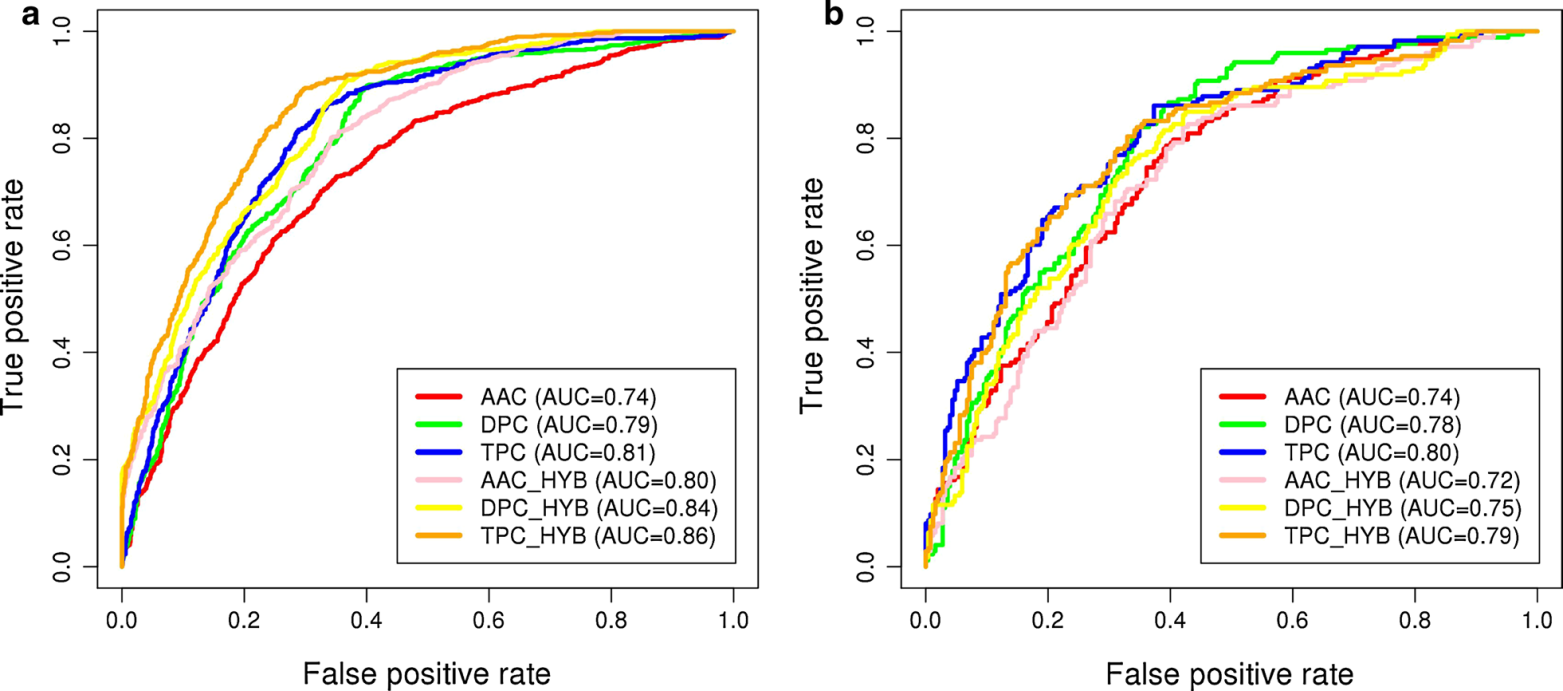
ROC plots of prediction models developed using SVM^light^ as machine learning technique; **a** 10-fold cross validation, **b** validation dataset

Compositional analysis revealed that AIEs and NAIEs show compositional differences. These characteristics were utilized to classify peptides into anti-inflammatory or non anti-inflammatory using machine learning models. SVM model with gamma parameter (g) = 0.005, trade-off factor (c) = 1 and cost factor (j) = 1 produced the best classification in AAC-based models. It showed MCC of 0.37, accuracy of 68.16% and the area under curve (AUC) was found to be 0.74 (Fig. 6).

#### Dipeptide composition-based models

Dipeptide composition was also used for building the predictive machine learning model using 400 sized vectors representing all possible dipeptides. SVM model with gamma parameter (g) = 0.005, trade-off factor (c) = 1 and cost factor (j) = 3 was the best performing model with an accuracy of 70.98, MCC of 0.44 and an area under curve (AUC) of 0.79 (Fig. 6).

#### Tripeptide composition-based models

The local order in the form of DPC as feature yielded better predictive ability of SVM model as compared to the results obtained using AAC. However, to further evaluate the effect of local order in the form of tripeptide, TPC as input feature was used to build the SVM model. The best model performed with an accuracy of 75.04%, MCC of 0.51 and AUC of 0.81 on gamma parameter (g) = 0.001, trade-off factor (c) = 4 and cost factor (j) = 1 (Fig. 6).

### Performance of RF models

RF models were constructed at optimized mtry and ntree for each input feature separately and performance of these models were evaluated using 10-fold cross validation and on the validation set (Additional file 1: Text S1). Performance of RF model was comparable to SVM models. For amino acid, dipeptide and selected tripeptide composition features SVM showed MCC of 0.37, 0.44 and 0.51, whereas, RF showed MCC of 0.39, 0.43 and 0.59 (Table 2 and Additional file 1: Table S2). The RF model constructed using selected tripeptide composition features displayed higher performance as compared to SVM, using 10-fold cross validation. However, on the validation set, the same RF model showed the MCC of 0.31 which is much lower as compared to the MCC (0.43) shown by SVM model (Table 3 and Additional file 1: Table S3). Hence, SVM models were implemented as the default model for the construction of hybrid models. Performance of RF models was also evaluated on the balanced dataset using 10-fold cross validation and on validation set (Additional file 1: Tables S4 and S5). Detailed description of machine learning comparison and RF models optimization and performance has been provided in Additional file 1: Text S1.

**Table 2:** Performance of SVM models using various sequence based features

Feature	Thre	Sen	Spe	Acc	MCC	Parameter
AAC	−0.3	71.74	65.71	68.16	0.37	g:0.005:c:1:j:1
DPC	−0.2	80.43	64.52	70.98	0.44	g:0.005:c:1:j:3
TPC	−0.2	79.28	72.15	75.04	0.51	g:0.001:c:4:j:1
AAC_HYB	−0.2	81.3	64.12	71.1	0.45	g:0.005 c:1 j:4
DPC_HYB	−0.3	84.78	67.2	74.34	0.51	g:0.005 c:1 j:1
TPC_HYB	−0.3	87.83	71.46	78.1	0.58	g:0.001 c:2 j:1

*Thre* threshold, *Sen* sensitivity, *Spe* specificity, *Acc* accuracy, *MCC* Matthews correlation coefficient

**Table 3:** Performance of optimized prediction models on validation dataset

Feature	Thre	Sen	Spe	Acc	MCC
AAC	−0.3	72.83	63.89	67.53	0.36
DPC	−0.2	81.5	65.08	71.76	0.46
TPC	−0.2	71.1	72.22	71.76	0.43
AAC_HYB	−0.2	72.83	62.3	66.59	0.35
DPC_HYB	−0.3	80.35	61.9	69.41	0.42
TPC_HYB	−0.3	78.61	67.46	72	0.45

*Thre* threshold, *Sen* sensitivity, *Spe* specificity, *Acc* accuracy, *MCC* Matthews correlation coefficient

### Hybrid model

TPC-based SVM model yielded the best accuracy among the three composition-based models. However, to further improve the efficiency of models, hybrid models were constructed for AAC, DPC, and TPC based models by combining each one with the motif features. The hybrid of AAC-based model (AAC_HYB) with motif features performed with 71.1% accuracy, whereas, the hybrid of DPC-based model (DPC_HYB) with motif features showed an accuracy of 74.34%. The hybrid of TPC-based model with motif features (TPC_HYB) was the best performing among all hybrid models. It showed the highest accuracy of 78.1%, MCC of 0.58 and an area under curve (AUC) of 0.86 (Fig. 6).

### Performance on validation dataset

The performance of all models was evaluated on the validation data (Table 3  and Fig. 6). This evaluation was carried out in addition to cross-validation to ensure that the obtained results were not due to over-optimization. TPC_HYB model which showed the highest accuracy in 10-fold cross validation also performed well on the validation dataset (72%). These results attest that the observed results were not due to over-optimization of data.

Furthermore, all the inputs such as amino acid, dipeptides, tripeptides etc., were processed for feature selection for composition and hybrid models by CfsSubsetEval algorithm of WEKA package. This feature selection is generally carried out to reduce the input data size without compromising on the performance. The final number of input features and performances of model with reduced input features are mentioned in Additional file 1: Table S6. It is apparent from Additional file 1: Table S7 that the performances of all models were reduced on validation dataset, with the reduction of feature vector size, however, in case of TPC_HYB model, the performance showed a marginal decline on the validation dataset, and thus, this model with reduced features was integrated in the webserver for efficient analysis.

In addition to 10-fold cross validation, models were also subjected to a fivefold cross validation analysis. The performance of models using fivefold cross validation also showed similar results. The TPC_HYB model displayed the maximum accuracy of 76% and also performed better than the other models for sensitivity and specificity values. It showed an MCC value of 0.56 and an AUC measure of 0.85 (Additional file 1: Table S8). Resultantly, the TPC_HYB model was incorporated in the webserver for epitope prediction.

Balanced dataset which contained 690 samples from both positive and negative dataset was also used to examine the performance of SVM models. The TPC_HYB model displayed the maximum accuracy of 77.8% and MCC value of 0.56. The sensitivity and specificity of this model was also higher than other models (Additional file 1: Table S9**)**.

### Web server and tools

A webserver was constructed for hosting a number of tools developed in this study for predicting the anti-inflammatory nature of peptide/protein antigens. These tools employ the validated best performing model developed in the study. The web server also incorporates several other analysis tools as described below.

### Peptide prediction

This module is developed for predicting the anti-inflammatory nature of small length peptides. The user can submit a single peptide or a batch of peptides with lengths ranging from 4 to 30 amino acids. A threshold option is also provided which can be tuned to increase the stringency of positive prediction. Queries are passed through anti-inflammatory epitope prediction pipeline and the results are displayed as a table which shows the prediction of a peptide as anti-inflammatory or non anti-inflammatory based on SVM or RF scores.

Every peptide sequence in the results table is provided with option for its virtual screening and design which is an additional feature that screens the variants of the peptides for anti-inflammatory property. Variants are created by substituting each amino acid position by all the 19 other naturally occurring amino acids. This option enables the user to check the anti-inflammatory nature of peptides that are similar to the query peptides.

### Protein scan

To determine the antigenic regions in a full-length protein which can induce anti-inflammatory responses, this module has been designed to allow the submission of query proteins with longer lengths. This tool divides the protein sequences into smaller peptides and runs through the same prediction pipeline. The user can also select the desired window length of peptides for prediction. Virtual screening option is also provided similar to the peptide prediction module.

### Epitope mapping

In addition to the above tools, the user may wish to map the experimentally validated anti-inflammatory antigenic regions on a query protein. This module map experimentally validated AIEs from IEDB database on the protein query sequence. The results are displayed as an alignment with links to the related assay provided by IEDB database.

### Similarity search

Unlike the epitope mapping tool, this module identifies the similar regions in the query peptide/protein and experimentally validated AIEs. Smith–Watermann search of query sequence in IEDB database is carried for displaying the top hits. Results are shown as alignments with links to related assay in IEDB database.

## Discussion

Anti-inflammatory drugs have widespread applications in the field of therapeutics because of their role in the reduction of swelling and inflammation. The novel peptides with anti-inflammatory properties may also be used as therapeutic agents against several diseases such as Alzheimer’s, rheumatoid arthritis as well as gut infections. Inflammatory response at the site of infection is caused due to the production of prostaglandin, thromboxane, etc. along with a release of histamine and kinin. Though it has been shown that nature of activating signal, timing, and sequence of cytokine action as well as experimental models affect their pro- or anti-inflammatory properties, IL4, IL-10, IL-13, IFN-alpha and transforming growth factor-beta are widely recognized as anti-inflammatory cytokines [30]. Studies have shown that anti-inflammatory drugs function by down-regulating the expressions of 5-LOX, COX-2, MMPs and NF-κB [31]. These drugs show low efficacy in curing the inflammation and leads to hypersensitivity reactions and deterioration of the immune system [32]. Due to the limitation of small molecule drugs, peptide-based drugs appear as better candidates in curing inflammation [33]. Thus, developing an in silico method for predicting the anti-inflammatory properties of newly synthesized or recently discovered amino acid sequence (peptide) is very valuable.

For the identification of anti-inflammatory targets, positive (anti-inflammatory) and negative (non anti-inflammatory) samples data was curated from immune epitope database which is a well-known reference database and contain the experimentally validated data. Using a good input quality data is a key for the application of machine learning techniques.

Compositional analysis revealed the presence of selected amino acids in abundance in AIEs, which is supported by other independent studies. For example, an amino acid stretch of 8-16 of SV-IV, which is rich in Ser and Gln residues, has been reported to exhibit anti-inflammatory properties [34]. Motif based analysis helped to determine the patterns in binding sites of target peptides on MHC molecules. Annotation of MERCI motifs from unique AIEs revealed that ‘hydrophobic hydrophobic tiny F charged’ had the maximum coverage. From this study, it was also observed that certain HLA alleles such as HLA-DRB1*04:01 alleles were more frequent in anti-inflammatory positive assays. Interestingly, the HLA-DRB1*04:01 allele has been associated with an increased occurrence of rheumatoid arthritis in the Caucasian population [35].

Furthermore, three different types of compositional analysis including amino acid, dipeptide and tripeptide composition were carried out to obtain the information of signature sequences, of which TPC_HYB (tripeptide hybrid with motifs derived weightage) was selected and included as the final model for webserver as a default prediction model. TPC (tripeptide composition) based RF model was also included at the webserver as an additional prediction model. The availability of two machine learning models provides flexibility and options to the users to predict epitopes using two different methods.

## Conclusion

The inherent ability of anti-inflammatory molecules to induce anti-inflammatory cytokines has the potential to play a major role in immunology and peptide therapeutic applications. Hence, an attempt has been made to identify sequence-based signatures of anti-inflammatory peptides and a machine learning tool has been developed for the prediction of anti-inflammatory epitopes. The observed accuracy values attest the performance of the tool and to our knowledge, it is the only available tool for the prediction of anti-inflammatory epitopes. Thus, the identification of anti-inflammatory target peptide sequences using this computational server provides valuable leads for experimental validations for the scientific community. The developed computational tools for investigation of anti-inflammatory sites have been made freely available for academic usage as a web server.

## Additional files

**Additional file 1.** Additional tables.

**Additional file 2: Figure S1** Comparison of machine learning algorithms using amino acid composition as feature input at fivefold cross validation.

**Additional file 3: Figure S2.** Comparison of machine learning algorithms using amino acid composition as feature input at ten-fold cross validation.

**Additional file 4: Figure S3** Optimization of mtry for random forest model using amino acid composition as feature input.

**Additional file 5: Figure S4.** Optimization of mtry and ntree for random forest model using amino acid composition as feature input.

**Additional file 6: Figure S5.** Performance of model constructed using amino acid composition as feature input at optimized parameters (mtry=2, ntree=2k) on validation set.

**Additional file 7: Figure S6.** Optimization of mtry for random forest model using dipeptide composition as feature input.

**Additional file 8: Figure S7.** Optimization of mtry and ntree for random forest model using dipeptide composition as feature input.

**Additional file 9: Figure S8.** Performance of model constructed using dipeptide composition as feature input at optimized parameters (mtry=11, ntree=2k) on validation set.

**Additional file 10: Figure S9.** Optimization of mtry for random forest model using tripeptide composition as feature input.

**Additional file 11: Figure S10.** Optimization of ntree for random forest model using tripeptide composition as feature input.

**Additional file 12: Figure S11.** Performance of model constructed using tripeptide composition as feature input at optimized parameters (mtry=24, ntree=1.5k) on validation set.

## Abbreviations

AAC: amino acid composition
ACC: accuracy
AIEs: anti-inflammatory epitopes
AUC: area under curve
DPC: dipeptide composition
FN: false negative
FP: false positive
IEDB: immune epitope database
HLA: human leukocyte antigen
MCC: Matthews correlation coefficient
MHC: major histocompatibility factor
NAIEs: non anti-inflammatory epitopes
SEN: sensitivity
SPC: specificity
SVM: support vector machine
TN: true negative
TP: true positive
TSL: two sample logo
TPC: tripeptide composition
RF: random forest
KNN: *k*-nearest neighbors
LDA: linear discriminant analysis
CART: classification and regression tree

## References

[1] Sun S-C, Chang J-H, Jin J (2013). Regulation of nuclear factor-κB in autoimmunity. Trends Immunol.

[2] Gonzalez-Rey E, Anderson P, Delgado M (2007). Emerging roles of vasoactive intestinal peptide: a new approach for autoimmune therapy. Ann Rheumc Dis..

[3] Delgado M, Ganea D (2008). Anti-inflammatory neuropeptides: a new class of endogenous immunoregulatory agents. Brain Behav Immun.

[4] Weiner HL, Lemere CA, Maron R, Spooner ET, Grenfell TJ, Mori C, Issazadeh S, Hancock WW, Selkoe DJ (2000). Nasal administration of amyloid-β peptide decreases cerebral amyloid burden in a mouse model of Alzheimer’s disease. Ann Neurol.

[5] Delgado M, Abad C, Martinez C, Leceta J, Gomariz RP (2001). Vasoactive intestinal peptide prevents experimental arthritis by downregulating both autoimmune and inflammatory components of the disease. Nat Med.

[6] Downer EJ, Cowley TR, Cox F, Maher FO, Berezin V, Bock E, Lynch MA (2009). A synthetic NCAM-derived mimetic peptide, FGL, exerts anti-inflammatory properties via IGF-1 and interferon-γ modulation. J Neurochem.

[7] Zouki C, Ouellet S, Filep JG (2000). The anti-inflammatory peptides, antiflammins, regulate the expression of adhesion molecules on human leukocytes and prevent neutrophil adhesion to endothelial cells. FASEB J.

[8] Gonzalez RR, Fong T, Belmar N, Saban M, Felsen D, Te A (2005). Modulating bladder neuro-inflammation: RDP58, a novel anti-inflammatory peptide, decreases inflammation and nerve growth factor production in experimental cystitis. J Urol.

[9] Bielekova B, Martin R (2001). Antigen-specific immunomodulation via altered peptide ligands. J Mol Med.

[10] Zhang Q, Wang P, Kim Y, Haste-Andersen P, Beaver J, Bourne PE, Bui H-H, Buus S, Frankild S, Greenbaum J (2008). Immune epitope database analysis resource (IEDB-AR). Nucleic Acids Res.

[11] Marie C, Pitton C, Fitting C, Cavaillon J (1996). Regulation by anti-inflammatory cytokines (IL-4, IL-10, IL-13, TGFβ) of interleukin-8 production by LPS-and/or TNFα-activated human polymorphonuclear cells. Mediat Inflamm.

[12] Gupta A, Kapil R, Dhakan DB, Sharma VK (2014). MP3: a software tool for the prediction of pathogenic proteins in genomic and metagenomic data. PLoS ONE.

[13] Sharma AK, Gupta A, Kumar S, Dhakan DB, Sharma VK (2015). Woods: a fast and accurate functional annotator and classifier of genomic and metagenomic sequences. Genomics.

[14] Zhang J, Zhao X, Sun P, Gao B, Ma Z: Conformational B-cell epitopes prediction from sequences using cost-sensitive ensemble classifiers and spatial clustering. BioMed Res Int. 2014;2014. 10.1155/2014/689219 PMC4083607 25045691

[15] Gupta S, Ansari HR, Gautam A, Raghava GP (2013). Identification of B-cell epitopes in an antigen for inducing specific class of antibodies. Biol Direct.

[16] Chaudhuri R, Ansari FA, Raghunandanan MV, Ramachandran S (2011). FungalRV: adhesin prediction and immunoinformatics portal for human fungal pathogens. BMC Genom.

[17] Sirskyj D, Diaz-Mitoma F, Golshani A, Kumar A, Azizi A (2011). Innovative bioinformatic approaches for developing peptide-based vaccines against hypervariable viruses. Immunol Cell Biol.

[18] Petrovsky N, Brusic V (2002). Computational immunology: the coming of age. Immunol Cell Biol.

[19] Gupta S, Madhu MK, Sharma AK, Sharma VK (2016). ProInflam: a webserver for the prediction of proinflammatory antigenicity of peptides and proteins. J Transl Med.

[20] Vens C, Rosso M-N, Danchin EG (2011). Identifying discriminative classification-based motifs in biological sequences. Bioinformatics.

[21] Rammensee H-G, Friede T, Stevanović S (1995). MHC ligands and peptide motifs: first listing. Immunogenetics.

[22] Nielsen M, Lund O, Buus S, Lundegaard C (2010). MHC class II epitope predictive algorithms. Immunology.

[23] Dhanda SK, Gupta S, Vir P, Raghava G: Prediction of IL4 inducing peptides. Clin Dev Immunol. 2013;2013. 10.1155/2013/263952 PMC3893860 24489573

[24] Dhanda SK, Vir P, Raghava GP (2013). Designing of interferon-gamma inducing MHC class-II binders. Biol Direct.

[25] Wang HW, Pai TW. Machine learning-based methods for prediction of linear B-cell epitopes. Immunoinformatics. 2014:217–36. 10.1007/978-1-4939-1115-8_12 25048127

[26] Liu Y (2004). Active learning with support vector machine applied to gene expression data for cancer classification. J Chem Info Comput Sci.

[27] Mohammad TAS, Nagarajaram HA (2011). Svm-based method for protein structural class prediction using secondary structural content and structural information of amino acids. J Bioinform Comput Biol.

[28] Zavaljevski N, Stevens FJ, Reifman J (2002). Support vector machines with selective kernel scaling for protein classification and identification of key amino acid positions. Bioinformatics.

[29] Saito T, Rehmsmeier M (2015). The precision-recall plot is more informative than the ROC plot when evaluating binary classifiers on imbalanced datasets. PLoS ONE.

[30] Cavaillon J-M (2001). Pro-versus anti-inflammatory cytokines: myth or reality. Cell Mol Biol.

[31] Vidal C, Gomez-Hernandez A, Sanchez-Galan E, Gonzalez A, Ortega L, Gomez-Gerique JA, Tunon J, Egido J (2007). Licofelone, a balanced inhibitor of cyclooxygenase and 5-lipoxygenase, reduces inflammation in a rabbit model of atherosclerosis. J Pharmacol Exp Ther.

[32] Janeway CA, Travers P, Walport M, Shlomchik MJ. The immune system in health and disease. 2001

[33] Veljaca M (2001). Anti-inflammatory peptides and proteins in inflammatory bowel disease. Curr Opinion Investig Drug..

[34] Ialenti A, Santagada V, Caliendo G, Severino B, Fiorino F, Maffia P, Ianaro A, Morelli F, Di Micco B, Cartenì M (2001). Synthesis of novel anti-inflammatory peptides derived from the amino-acid sequence of the bioactive protein SV-IV. Eur J Biochem.

[35] Fries JF, Wolfe F, Apple R, Erlich H, Bugawan T, Holmes T, Bruce B (2002). HLA–DRB1 genotype associations in 793 white patients from a rheumatoid arthritis inception cohort: frequency, severity, and treatment bias. Arthritis Rheum.

