# Supplementary material for: Prediction of anti-inflammatory proteins/peptides: an insilico approach
Source: J Transl Med. 2017 Jan 6;15:7. doi: 10.1186/s12967-016-1103-6 (PMC5216551; doi:10.1186/s12967-016-1103-6)
Supplement: Supplementary file 1 — Additional file 1. Additional tables. [file 12967_2016_1103_MOESM1_ESM.doc]

**Text S1. Comparison of various machine learning methods and optimization of Random Forest**

**Amino acid composition**

Different machine learning approaches have been implemented using caret package in R. Amino acid frequency has been used as input feature for the evaluation of these approaches via 5-folds and 10-folds cross validation methods. The performance of RF and SVM was higher in comparison to other machine learning approaches. For 5-folds cross validation, ROC values were 0.79 and 0.72, and for 10-folds cross validation ROC values were 0.80 and 0.72 for RF and SVM models, respectively (Supplementary Figure S1 and S2).

Optimization of RF was carried out using random forest package using amino acid composition feature as input. The parameters such as mtry and ntree have been optimized and selected for the model construction on the basis of their performance (Supplementary Figure S3 and S4). RF model with parameters such as mtry = 2, ntree = 2000 using 10-folds cross validation showed accuracy of 0.71 and MCC of 0.39, whereas, on the validation set it showed an accuracy of 0.71 and MCC of 0.39 (Supplementary Table S2 and 3). The performance of optimized model on validation set is provided in Supplementary Figure S5.

**Dipeptide composition**

Optimization of RF was carried out using random forest package using dipeptide composition feature as input. The parameters such as mtry and ntree have been optimized and selected for the model construction on the basis of their performance (Supplementary Figure S6 and S7). RF model with parameters such as mtry = 11, ntree = 2000 using 10-folds cross validation showed accuracy of 0.73 and MCC of 0.43, whereas, on the validation set it showed an accuracy of 0.70 and MCC of 0.38 (Supplementary Table S2 and S3). The performance of the optimized model on validation set is provided in Supplementary Figure S8.

**Tripeptide composition**

Optimization of RF was carried out using random forest package using 276 selected tripeptide composition features as input. The mtry was optimized using tuneRF function (Supplementary Figure S9). The optimized mtry =24 (less OOB) was used to optimize the ntree (Supplementary Figure S10). RF model with parameters such as mtry = 24, ntree = 1500 using 10-folds cross validation showed an accuracy of 0.77 and MCC of 0.56, whereas, on the validation set it showed an accuracy of 0.63 and MCC of 0.31 (Supplementary Table S2 and S3). The performance of optimized model on validation set has been provided in Supplementary Figure S11.

The above performances have been evaluated on the real dataset. However, these performances using amino acid, dipeptide and tripeptide composition features were also evaluated for the balanced datasets using 10-fold cross validation and on validation set (Supplementary Table S4 and S5).

**Table S1.** Tripeptide composition (TPC) analysis of AIEs and Non-AIEs. Only significant tripeptides (p value <=0.01) are shown.

| **Tripeptides** | **TPC AIEs** | **TPC Non-AIEs** | **Standard error (AIEs)** | **Standard error (Non-AIEs)** | **t.test** |
| --- | --- | --- | --- | --- | --- |
| **AAA** | **0.052** | **0.265** | **0.023** | **0.054** | **0.0003** |
| **AAG** | **0** | **0.16** | **0** | **0.038** | **0** |
| **AAK** | **0** | **0.094** | **0** | **0.026** | **0.0003** |
| **AAP** | **0.008** | **0.145** | **0.008** | **0.036** | **0.0002** |
| **AAT** | **0.041** | **0.223** | **0.021** | **0.054** | **0.0017** |
| **AFS** | **0** | **0.052** | **0** | **0.02** | **0.0083** |
| **AGK** | **0** | **0.062** | **0** | **0.021** | **0.0029** |
| **AGT** | **0** | **0.1** | **0** | **0.027** | **0.0002** |
| **AIT** | **0** | **0.053** | **0** | **0.02** | **0.0081** |
| **AKY** | **0** | **0.061** | **0** | **0.021** | **0.0046** |
| **APA** | **0.007** | **0.107** | **0.007** | **0.03** | **0.0013** |
| **ARL** | **0.125** | **0** | **0.045** | **0** | **0.0062** |
| **ATA** | **0.023** | **0.151** | **0.014** | **0.044** | **0.0055** |
| **ATP** | **0.056** | **0.222** | **0.026** | **0.045** | **0.0014** |
| **ATT** | **0.036** | **0.13** | **0.018** | **0.031** | **0.0094** |
| **ATV** | **0.011** | **0.112** | **0.011** | **0.03** | **0.0019** |
| **ATY** | **0** | **0.053** | **0** | **0.02** | **0.0081** |
| **AVV** | **0** | **0.109** | **0** | **0.028** | **0.0001** |
| **CGR** | **0** | **0.047** | **0** | **0.018** | **0.0089** |
| **DDN** | **0** | **0.1** | **0** | **0.029** | **0.0006** |
| **DEP** | **0** | **0.059** | **0** | **0.021** | **0.0048** |
| **DEQ** | **0** | **0.052** | **0** | **0.02** | **0.0082** |
| **DKW** | **0** | **0.053** | **0** | **0.02** | **0.0081** |
| **DNG** | **0** | **0.103** | **0** | **0.03** | **0.0006** |
| **DNT** | **0** | **0.072** | **0** | **0.024** | **0.0028** |
| **DPD** | **0** | **0.077** | **0** | **0.026** | **0.0028** |
| **EEQ** | **0** | **0.092** | **0** | **0.026** | **0.0006** |
| **EKL** | **0.061** | **0** | **0.024** | **0** | **0.0093** |
| **ENQ** | **0.066** | **0** | **0.025** | **0** | **0.0089** |
| **EQK** | **0.009** | **0.118** | **0.009** | **0.029** | **0.0004** |
| **FAV** | **0.016** | **0.099** | **0.011** | **0.027** | **0.0041** |
| **FTV** | **0.023** | **0.122** | **0.016** | **0.03** | **0.0042** |
| **GAA** | **0.026** | **0.175** | **0.015** | **0.043** | **0.0012** |
| **GAG** | **0.025** | **0.151** | **0.014** | **0.033** | **0.0004** |
| **GAT** | **0.006** | **0.095** | **0.006** | **0.026** | **0.001** |
| **GHD** | **0** | **0.069** | **0** | **0.023** | **0.0027** |
| **GIM** | **0** | **0.053** | **0** | **0.02** | **0.0081** |
| **GKA** | **0.03** | **0.117** | **0.017** | **0.029** | **0.0099** |
| **GKT** | **0** | **0.051** | **0** | **0.019** | **0.0084** |
| **GKV** | **0** | **0.055** | **0** | **0.021** | **0.0081** |
| **GNF** | **0** | **0.053** | **0** | **0.02** | **0.0081** |
| **GPQ** | **0** | **0.082** | **0** | **0.025** | **0.0009** |
| **GPT** | **0** | **0.051** | **0** | **0.019** | **0.0083** |
| **GRH** | **0** | **0.04** | **0** | **0.015** | **0.0081** |
| **GSS** | **0.118** | **0.017** | **0.035** | **0.01** | **0.0063** |
| **GTK** | **0** | **0.051** | **0** | **0.019** | **0.0084** |
| **HDK** | **0** | **0.052** | **0** | **0.02** | **0.0081** |
| **HDP** | **0** | **0.039** | **0** | **0.015** | **0.0081** |
| **HHL** | **0** | **0.053** | **0** | **0.019** | **0.0051** |
| **HLA** | **0** | **0.053** | **0** | **0.02** | **0.0081** |
| **ILS** | **0.083** | **0.006** | **0.028** | **0.006** | **0.0075** |
| **IPK** | **0.01** | **0.087** | **0.01** | **0.025** | **0.0041** |
| **ISN** | **0.139** | **0.013** | **0.044** | **0.009** | **0.0051** |
| **KGK** | **0** | **0.061** | **0** | **0.021** | **0.0046** |
| **KGL** | **0.07** | **0** | **0.027** | **0** | **0.0095** |
| **KGN** | **0** | **0.072** | **0** | **0.023** | **0.0017** |
| **KLD** | **0** | **0.066** | **0** | **0.022** | **0.0028** |
| **KLI** | **0.03** | **0.125** | **0.017** | **0.03** | **0.0064** |
| **KPE** | **0.008** | **0.075** | **0.008** | **0.023** | **0.0056** |
| **KPT** | **0** | **0.058** | **0** | **0.02** | **0.0048** |
| **KVT** | **0** | **0.061** | **0** | **0.021** | **0.0046** |
| **LAF** | **0** | **0.066** | **0** | **0.024** | **0.0056** |
| **LDN** | **0** | **0.039** | **0** | **0.015** | **0.0081** |
| **LEN** | **0.145** | **0.014** | **0.039** | **0.01** | **0.0013** |
| **LGA** | **0.016** | **0.086** | **0.011** | **0.024** | **0.0079** |
| **LKL** | **0.141** | **0.019** | **0.037** | **0.014** | **0.002** |
| **LRE** | **0** | **0.051** | **0** | **0.02** | **0.0093** |
| **LRL** | **0.106** | **0.006** | **0.036** | **0.006** | **0.0057** |
| **LVC** | **0.094** | **0** | **0.035** | **0** | **0.0076** |
| **NDK** | **0.006** | **0.066** | **0.006** | **0.02** | **0.0044** |
| **NIT** | **0** | **0.084** | **0** | **0.025** | **0.0009** |
| **NPE** | **0** | **0.05** | **0** | **0.018** | **0.0066** |
| **NTD** | **0** | **0.094** | **0** | **0.026** | **0.0003** |
| **PAD** | **0.018** | **0.107** | **0.013** | **0.028** | **0.0045** |
| **PAT** | **0.014** | **0.122** | **0.01** | **0.032** | **0.0014** |
| **PDN** | **0.019** | **0.132** | **0.014** | **0.033** | **0.0017** |
| **PEG** | **0.009** | **0.073** | **0.009** | **0.023** | **0.01** |
| **PGD** | **0** | **0.057** | **0** | **0.022** | **0.0088** |
| **PGP** | **0.019** | **0.175** | **0.013** | **0.04** | **0.0002** |
| **PKV** | **0** | **0.094** | **0** | **0.025** | **0.0002** |
| **PLL** | **0.102** | **0.012** | **0.032** | **0.009** | **0.0061** |
| **PLT** | **0** | **0.066** | **0** | **0.022** | **0.0028** |
| **PPG** | **0.03** | **0.165** | **0.018** | **0.038** | **0.0012** |
| **PPP** | **0** | **0.169** | **0** | **0.062** | **0.0065** |
| **PPS** | **0** | **0.062** | **0** | **0.023** | **0.0064** |
| **PSP** | **0** | **0.043** | **0** | **0.017** | **0.0088** |
| **PTA** | **0.007** | **0.112** | **0.007** | **0.029** | **0.0004** |
| **QAY** | **0** | **0.076** | **0** | **0.024** | **0.0015** |
| **QKL** | **0.019** | **0.107** | **0.014** | **0.028** | **0.0055** |
| **QSI** | **0** | **0.066** | **0** | **0.022** | **0.0028** |
| **RRF** | **0** | **0.039** | **0** | **0.015** | **0.0081** |
| **SEA** | **0.008** | **0.105** | **0.008** | **0.028** | **0.0009** |
| **SIP** | **0** | **0.065** | **0** | **0.022** | **0.0028** |
| **SLS** | **0.131** | **0.03** | **0.035** | **0.014** | **0.0066** |
| **SSK** | **0.032** | **0.136** | **0.016** | **0.032** | **0.0034** |
| **STW** | **0.008** | **0.076** | **0.008** | **0.024** | **0.0072** |
| **SVI** | **0.168** | **0.019** | **0.049** | **0.014** | **0.0037** |
| **TAA** | **0.018** | **0.126** | **0.013** | **0.03** | **0.0012** |
| **TAT** | **0.022** | **0.127** | **0.016** | **0.036** | **0.0077** |
| **TDD** | **0.009** | **0.119** | **0.009** | **0.03** | **0.0004** |
| **TEE** | **0.042** | **0.154** | **0.019** | **0.034** | **0.0036** |
| **TGA** | **0.011** | **0.097** | **0.011** | **0.027** | **0.0032** |
| **TIG** | **0** | **0.052** | **0** | **0.02** | **0.0082** |
| **TPA** | **0.014** | **0.136** | **0.01** | **0.04** | **0.0031** |
| **TPE** | **0.038** | **0.152** | **0.019** | **0.034** | **0.0035** |
| **TTE** | **0** | **0.124** | **0** | **0.031** | **0.0001** |
| **VDI** | **0** | **0.077** | **0** | **0.024** | **0.0011** |
| **VKP** | **0.014** | **0.102** | **0.014** | **0.027** | **0.0045** |
| **VLD** | **0.01** | **0.106** | **0.01** | **0.027** | **0.0008** |
| **VLE** | **0.103** | **0.006** | **0.033** | **0.006** | **0.0039** |
| **VLF** | **0** | **0.079** | **0** | **0.026** | **0.0023** |
| **VPK** | **0** | **0.068** | **0** | **0.023** | **0.0028** |
| **VPP** | **0.026** | **0.139** | **0.016** | **0.033** | **0.0021** |
| **VTF** | **0** | **0.053** | **0** | **0.02** | **0.0081** |
| **VTI** | **0** | **0.053** | **0** | **0.02** | **0.0081** |
| **VVA** | **0.029** | **0.145** | **0.021** | **0.032** | **0.0028** |
| **VWG** | **0** | **0.061** | **0** | **0.021** | **0.0046** |
| **WEQ** | **0** | **0.061** | **0** | **0.021** | **0.0046** |
| **YAA** | **0** | **0.052** | **0** | **0.02** | **0.0082** |
| **YAP** | **0** | **0.049** | **0** | **0.019** | **0.0086** |
| **YGK** | **0** | **0.054** | **0** | **0.019** | **0.005** |
| **YTT** | **0** | **0.057** | **0** | **0.02** | **0.005** |

**Table S2.** Performance of optimized RF prediction models using ten-fold cross validation.

TPC_Fsel* = Selected tripeptide composition features

| **Feature** | **Sensitivity** | **Specificity** | **Accuracy** | **MCC** | **Parameters** |
| --- | --- | --- | --- | --- | --- |
| **AAC** | 0.69 | 0.73 | 0.71 | 0.39 | mtry=2, ntree=2000 |
| **DPC** | 0.71 | 0.74 | 0.73 | 0.43 | mtry=11, ntree=2000 |
| **TPC_Fsel** | 0.89 | 0.77 | 0.8 | 0.59 | mtry=24, ntree=1500 |

**Table S3.** Performance of optimized RF prediction models on validation set.

| Feature | Sensitivity | Specificity | Accuracy | MCC | Parameters |
| --- | --- | --- | --- | --- | --- |
| AAC | 0.51 | 0.85 | 0.71 | 0.39 | mtry=2, ntree=2000 |
| DPC | 0.53 | 0.83 | 0.71 | 0.38 | mtry=11, ntree=2000 |
| TPC_Fsel | 0.37 | 0.89 | 0.68 | 0.31 | mtry=24, ntree=1500 |

TPC_Fsel* = Selected tripeptide composition features

**Table S4.** Performance of optimized RF prediction models using ten-fold cross validation on balanced dataset.

| Feature | Sensitivity | Specificity | Accuracy | MCC | Parameters |
| --- | --- | --- | --- | --- | --- |
| AAC | 0.72 | 0.73 | 0.72 | 0.44 | mtry=2, ntree=2000 |
| DPC | 0.75 | 0.7 | 0.72 | 0.45 | mtry=11, ntree=2000 |
| TPC_Fsel | 0.71 | 0.86 | 0.77 | 0.56 | mtry=24, ntree=1500 |

TPC_Fsel* = Selected tripeptide composition features

**Table S5.** Performance of optimized RF prediction models on validation set using balanced dataset.

| Feature | Sensitivity | Specificity | Accuracy | MCC | Parameters |
| --- | --- | --- | --- | --- | --- |
| AAC | 0.69 | 0.71 | 0.7 | 0.39 | mtry=2, ntree=2000 |
| DPC | 0.61 | 0.78 | 0.71 | 0.39 | mtry=11, ntree=2000 |
| TPC_Fsel | 0.79 | 0.53 | 0.63 | 0.31 | mtry=24, ntree=1500 |

TPC_Fsel* = Selected tripeptide composition features

**Table S6.** Performance of SVM models using various sequence based features after feature selection.

| **Feature** | **Thre.** | **Sen** | **Spe.** | **Acc.** | **MCC** | **Parameter** | **Number of features** |
| --- | --- | --- | --- | --- | --- | --- | --- |
| **AAC** | -0.3 | 62.9 | 71.06 | 67.75 | 0.34 | 0.7 | 13 |
| **DPC** | -0.1 | 70.43 | 61.55 | 65.16 | 0.31 | 0.68 | 41 |
| **TPC** | -0.7 | 81.16 | 75.42 | 77.75 | 0.56 | 0.89 | 276 |
| **AAC_HYB** | -0.3 | 68.84 | 74.03 | 71.92 | 0.42 | 0.77 | 13 |
| **DPC_HYB** | -0.9 | 75.36 | 65.51 | 69.51 | 0.4 | 0.78 | 41 |
| **TPC_HYB** | -0.6 | 71.88 | 90.49 | 82.93 | 0.64 | 0.89 | 276 |

Thre: Threshold; Sen: Sensitivity; Spe: Specificity; Acc: Accuracy; MCC: Matthews Correlation Coefficient

**Table S7.** Performance of optimized prediction models on validation dataset after feature selection.

| **Feature** | **Thre.** | **Sen** | **Spe.** | **Acc.** | **MCC** | **AUC** |
| --- | --- | --- | --- | --- | --- | --- |
| **AAC** | -0.3 | 56.07 | 68.25 | 63.29 | 0.24 | 0.67 |
| **DPC** | -0.1 | 58.96 | 60.32 | 59.76 | 0.19 | 0.61 |
| **TPC** | -0.7 | 43.35 | 83.33 | 67.06 | 0.29 | 0.72 |
| **AAC_HYB** | -0.3 | 57.8 | 69.44 | 64.71 | 0.27 | 0.68 |
| **DPC_HYB** | -0.9 | 78.03 | 45.63 | 58.82 | 0.24 | 0.64 |
| **TPC_HYB** | -0.6 | 45.66 | 82.54 | 67.53 | 0.31 | 0.73 |

It should be noted that the input vector size in tripeptide-composition-based feature was 8,000 due to which the model is likely to perform slower for large number of sequences. Therefore, feature selection, shown above, was performed on all the prediction models (AAC, DPC, TPC & their hybrid with motifs). The feature selection marginally reduced the accuracy on validation set, however, due to the considerable reduction in the analysis time on feature selection, the TPC_HYB model with selected features was considered for integration on the web server.

**Table S8.** Performance of models using five-fold cross validation.

| **Feature** | **Thre.** | **Sen** | **Spe.** | **Acc.** | **MCC** | **AUC** | **Parameter** |
| --- | --- | --- | --- | --- | --- | --- | --- |
| **AAC** | -0.3 | 70.29 | 67.1 | 68.39 | 0.37 | 0.73 | g:0.005:c:1:j:1 |
| **DPC** | -0.2 | 77.1 | 66.3 | 70.69 | 0.43 | 0.79 | g:0.005:c:80:j:5 |
| **TPC** | -0.4 | 86.23 | 63.92 | 72.98 | 0.5 | 0.81 | g:0.001:c:1:j:1 |
| **AAC_HYB** | -0.2 | 80.72 | 64.82 | 71.28 | 0.45 | 0.8 | g:0.01 c:2 j:2 |
| **DPC_HYB** | -0.2 | 81.74 | 67.79 | 73.45 | 0.49 | 0.83 | g:0.005 c:1 j:3 |
| **TPC_HYB** | -0.3 | 88.12 | 68.29 | 76.34 | 0.56 | 0.85 | g:0.001 c:2 j:1 |

**Table S9.** Performance of models using balanced dataset.

| **Feature** | **Thre.** | **Sen** | **Spe.** | **Acc.** | **MCC** | **AUC** | **Parameter** |
| --- | --- | --- | --- | --- | --- | --- | --- |
| **AAC** | 0 | 67.68 | 64.64 | 66.16 | 0.32 | 0.71 | g:0.001:c:1:j:1 |
| **DPC** | 0 | 91.01 | 54.06 | 72.54 | 0.49 | 0.5 | g:1:c:0.0005:j:1 |
| **TPC** | 0 | 85.22 | 61.59 | 73.41 | 0.48 | 0.8 | g:0.001:c:1:j:1 |
| **AAC_HYB** | 0 | 80.43 | 60.87 | 70.65 | 0.42 | 0.79 | g:0.01 c:1 j:2 |
| **DPC_HYB** | 0 | 93.91 | 55.8 | 74.86 | 0.54 | 0.83 | g:0.01 c:1 j:1 |
| **TPC_HYB** | 0.1 | 79.71 | 75.94 | 77.83 | 0.56 | 0.85 | g:0.001 c:1 j:1 |
