# Supplementary figures and images for: Prediction of anti-inflammatory proteins/peptides: an insilico approach
Source: J Transl Med. 2017 Jan 6;15:7. doi: 10.1186/s12967-016-1103-6 (PMC5216551; doi:10.1186/s12967-016-1103-6)

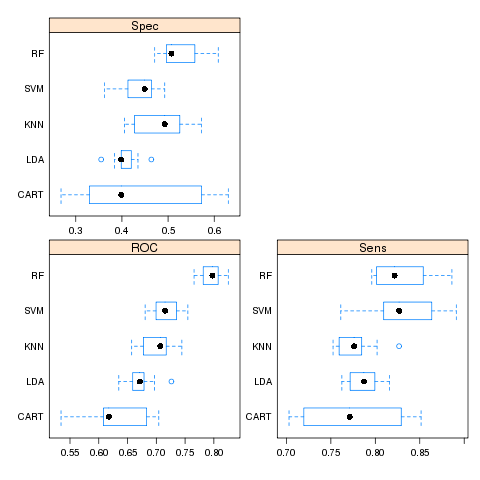

Supplement: Supplementary file 2 — Additional file 2: Figure S1 Comparison of machine learning algorithms using amino acid composition as feature input at fivefold cross validation. [file 12967_2016_1103_MOESM2_ESM.png]

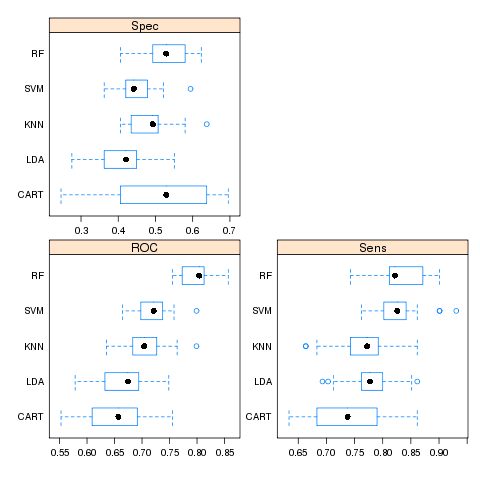

Supplement: Supplementary file 3 — Additional file 3: Figure S2. Comparison of machine learning algorithms using amino acid composition as feature input at ten-fold cross validation. [file 12967_2016_1103_MOESM3_ESM.png]

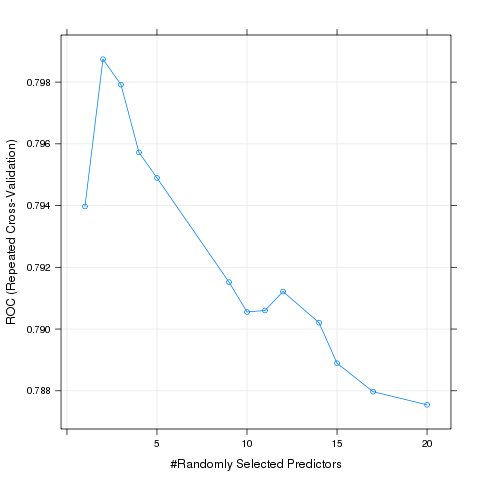

Supplement: Supplementary file 4 — Additional file 4: Figure S3 Optimization of mtry for random forest model using amino acid composition as feature input. [file 12967_2016_1103_MOESM4_ESM.png]

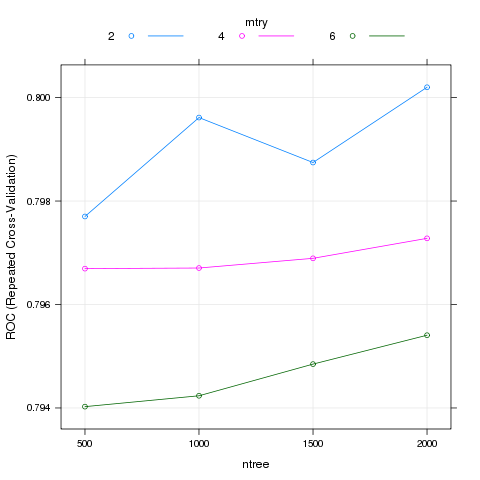

Supplement: Supplementary file 5 — Additional file 5: Figure S4. Optimization of mtry and ntree for random forest model using amino acid composition as feature input. [file 12967_2016_1103_MOESM5_ESM.png]

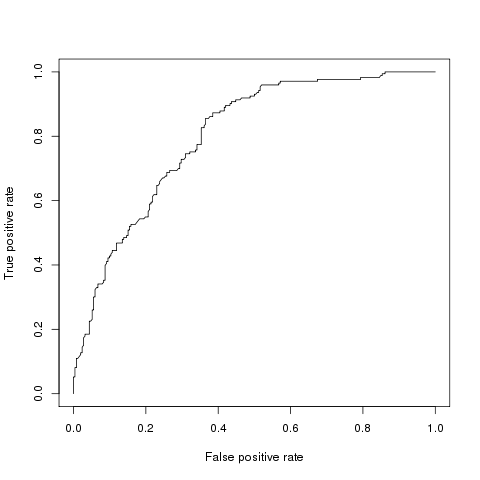

Supplement: Supplementary file 6 — Additional file 6: Figure S5. Performance of model constructed using amino acid composition as feature input at optimized parameters (mtry=2, ntree=2k) on validation set. [file 12967_2016_1103_MOESM6_ESM.png]

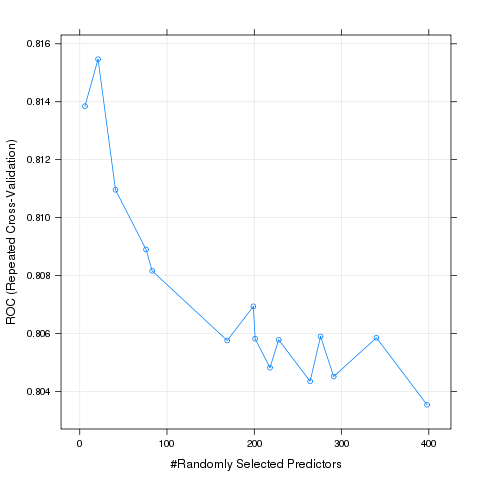

Supplement: Supplementary file 7 — Additional file 7: Figure S6. Optimization of mtry for random forest model using dipeptide composition as feature input. [file 12967_2016_1103_MOESM7_ESM.png]

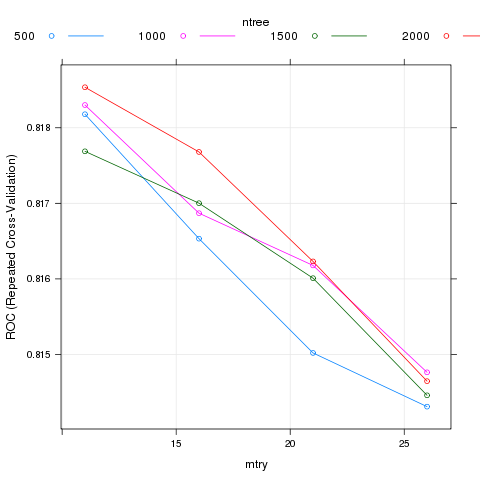

Supplement: Supplementary file 8 — Additional file 8: Figure S7. Optimization of mtry and ntree for random forest model using dipeptide composition as feature input. [file 12967_2016_1103_MOESM8_ESM.png]

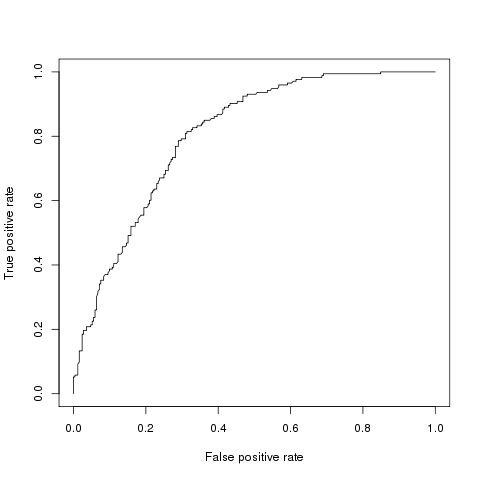

Supplement: Supplementary file 9 — Additional file 9: Figure S8. Performance of model constructed using dipeptide composition as feature input at optimized parameters (mtry=11, ntree=2k) on validation set. [file 12967_2016_1103_MOESM9_ESM.png]

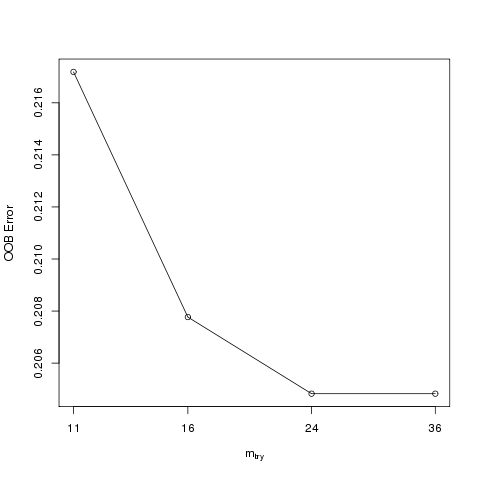

Supplement: Supplementary file 10 — Additional file 10: Figure S9. Optimization of mtry for random forest model using tripeptide composition as feature input. [file 12967_2016_1103_MOESM10_ESM.png]

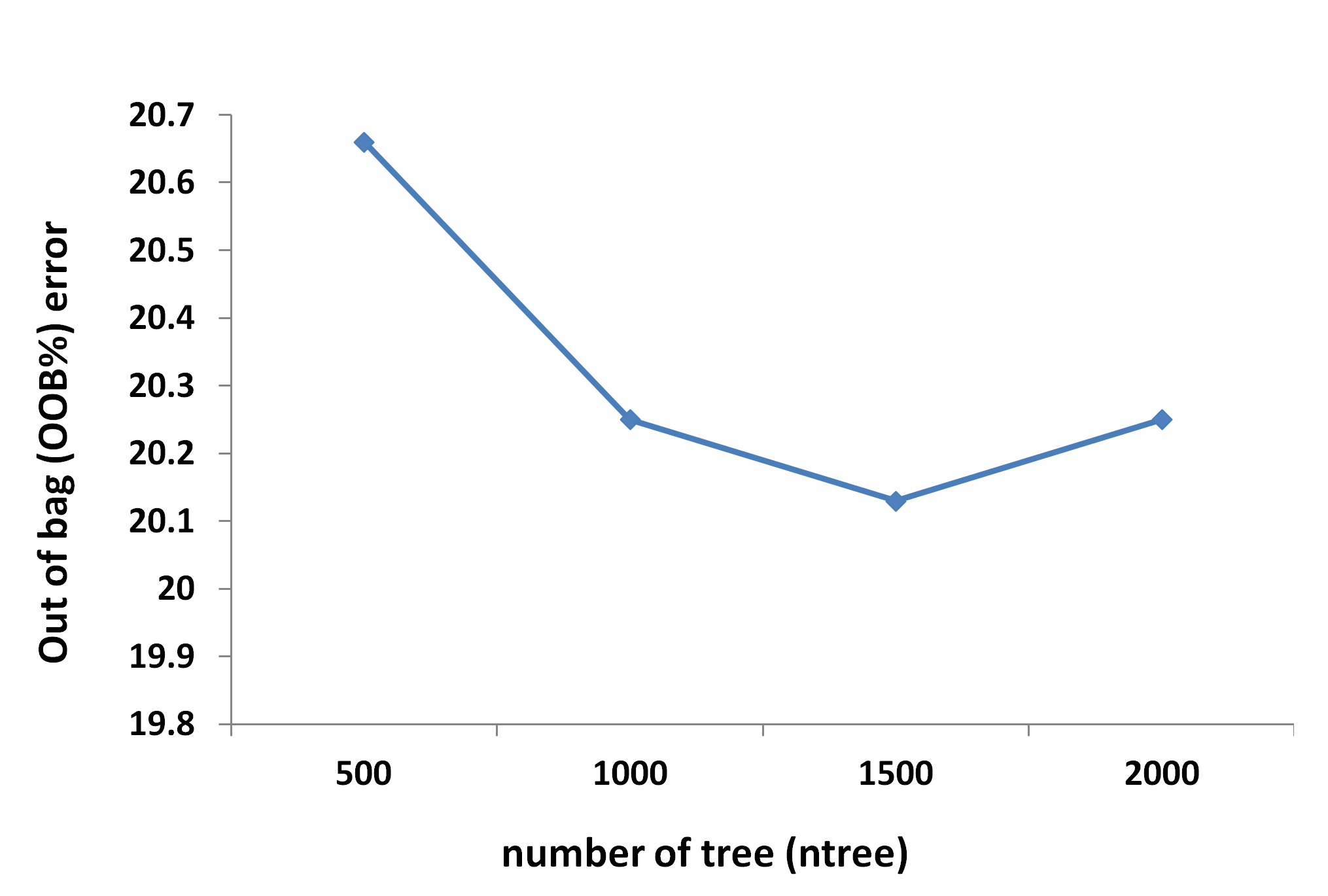

Supplement: Supplementary file 11 — Additional file 11: Figure S10. Optimization of ntree for random forest model using tripeptide composition as feature input. [file 12967_2016_1103_MOESM11_ESM.png]

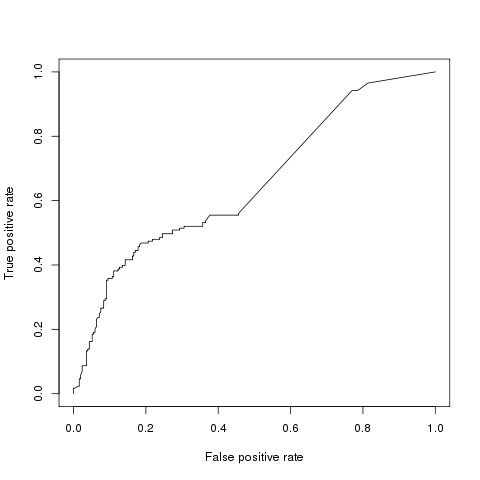

Supplement: Supplementary file 12 — Additional file 12: Figure S11. Performance of model constructed using tripeptide composition as feature input at optimized parameters (mtry=24, ntree=1.5k) on validation set. [file 12967_2016_1103_MOESM12_ESM.png]
